# Long-Term Risk Factors for Intracranial In-Stent Restenosis From a Multicenter Trial of Stenting for Symptomatic Intracranial Artery Stenosis Registry in China

**DOI:** 10.3389/fneur.2020.601199

**Published:** 2021-01-26

**Authors:** Xu Guo, Ning Ma, Feng Gao, Da-Peng Mo, Gang Luo, Zhong-Rong Miao

**Affiliations:** Department of Interventional Neuroradiology, Beijing Tiantan Hospital, Capital Medical University, Beijing, China

**Keywords:** cerebrovascular disease, stroke, endovascular treatment, interventional neurology, intracranial in-stent restenosis

## Abstract

**Background:** For patients with symptomatic intracranial artery stenosis (sICAS), endovascular treatment has been shown to be feasible and safe in recent studies. However, in-stent restenosis (ISR) risks the recurrence of ischemic stroke. We attempt to elucidate the risk factors for ISR.

**Methods:** We retrospectively analyzed 97 patients with sICAS from a prospective registry trial that included 20 centers from September 2013 to January 2015. Cases were classified into the ISR≥ 50% group or the ISR < 50% group. The baseline characteristics and long-term follow-up were compared between the two groups. Binary logistic regression analyses were identified as an association between ISR and endovascular technique factors.

**Results:** According to whether ISR was detected by CT angiography, 97 patients were divided into the ISR group (*n* = 24) and the non-ISR group (*n* = 73). The admission baseline features and lesion angiography characteristics were similar, while plasma hs-CRP (mg/L) was higher in the ISR≥ 50% group at admission (8.2 ± 11.4 vs. 2.8 ± 4.1, *p* = 0.032). Binary logistic regression analysis identified the longer stents (adjusted OR 0.816, 95% CI 0.699–0.953; *p* = 0.010), balloon-mounted stents (adjusted OR 5.748, 95% CI 1.533–21.546; *p* = 0.009), and local anesthesia (adjusted OR 6.000, 95% CI 1.693–21.262; *p* = 0.006) as predictors of ISR at the 1-year follow-up.

**Conclusions:** The longer stents, balloon-mounted stents implanted in the intracranial vertebral or basilar artery, and local anesthesia were significantly associated with in-stent restenosis. Further studies are required to identify accurate biomarkers or image markers associated with ISR in ICAS patients.

**Clinical Trial Registration:** www.ClinicalTrials.gov, identifier: NCT01968122.

## Introduction

The prevalence of intracranial atherosclerotic stenosis (ICAS) in Chinese patients was up to 46.6% in symptomatic ischemic stroke patients (1). Symptomatic ICAS (sICAS) is associated with recurrent ischemic stroke (2). SAMMPRIS and VISSIT trials have shown that aggressive medical management has been more effective and safer than endovascular therapy in the past decade (3, 4). However, a recent Wingspan Stent System Post Market Surveillance Study (WEAVE) indicated that the perioperative complication rate is quite low for on-label patients (2.6%). Patients enrolled in this study, including patients with symptomatic and severe ICAS lesions, had suffered at least two ischemic strokes (5). It is obvious that patients with sICAS who failed the best medical treatment would benefit from endovascular therapy.

As we reported, the 30-days rate of primary endpoints, including stroke, transient ischemic attack, and death, was 4.3% in a multicenter prospective registry study of stenting for sICAS in China (6). The incidence of the composite endpoint in this study at 1 year was 8.1%, and restenosis ≥50% was found in 27.6% of patients at the 12-months follow-up. Although the majority of patients (78.9%) were asymptomatic (7), restenosis would be a risk factor for ischemic stroke, causing acute large vessel occlusion or transient ischemic attack (TIA) (2). Therefore, in the present study, according to the inflammatory index (hs-CRP), features of the lesion in angiography, and characteristics of the stent in the operation procedure, we aimed to identify risk factors for in-stent restenosis of endovascular treatment in intracranial atherosclerotic stenosis in a 12-months follow-up.

## Methods

### Overall Design

Details of the protocol of the Aire/Wire-China trial were published (8). This study was a new subgroup *post-hoc* analysis of a multicenter prospective non-randomized registry trial that included 20 participating centers from September 2013 to January 2015. Approval by each site's institutional review board or ethics committee was obtained. Written informed consent was required from each patient or their legally authorized representative. All reported endpoints were evaluated and confirmed by a central adjudication committee composed of designated neurologists, neurosurgeons, and radiologists blinded to the treatment choices. An independent Data and Safety Monitoring Board (DSMB) oversaw the conduction, safety, and efficacy of the study.

### Study Population

Inclusion and exclusion criteria were established by the executive committee. Patients were aged 18–85 years and had a symptomatic ICAS of 70–99% with a lesion length of ≤15 mm and a target vessel diameter of ≥2.0 mm in the intracranial internal carotid artery, middle cerebral artery, intracranial vertebral artery, or basilar artery. The measurements were made on digital subtraction angiography (DSA) using the warfarin–aspirin symptomatic intracranial disease method with normal distal vessels as the reference (9). The symptoms could be transient ischemic attack (TIA) or stroke onset within the past 3 months but had to be attributable to hypoperfusion in the territory of the target lesion. Hemodynamic impairment in the territory of the culprit artery was determined on imaging within 2 weeks before the operation using any one of the following methods: (1) Hypoperfusion in the target anterior or posterior circulation territory by computer tomography perfusion (CTP) or single-photon emission CT (SPECT); (2) An American Society of Interventional and Therapeutic Neuroradiology/Society of Interventional Radiology Collateral Flow Grading System score of <3 on DSA (10); (3) Hemodynamic ischemic lesion by magnetic resonance imaging (MRI); and (4) A peak systolic velocity of ≥200 cm/s and ≤1 collateral vessel that could be insonated on transcranial Doppler examination (11). In this study, hemodynamic ischemic lesions on MRI were defined as small ischemic infarcts in a watershed distribution in the culprit vessel territory (12). Lesions that could be entirely explained as an embolic phenomenon or lacunar infarcts were excluded from this category. The images were centrally reviewed by at least two physicians who were allowed to resolve the disagreement through discussion. The patients were excluded from the study if the raters could not agree on the classification.

**Figure 1:**
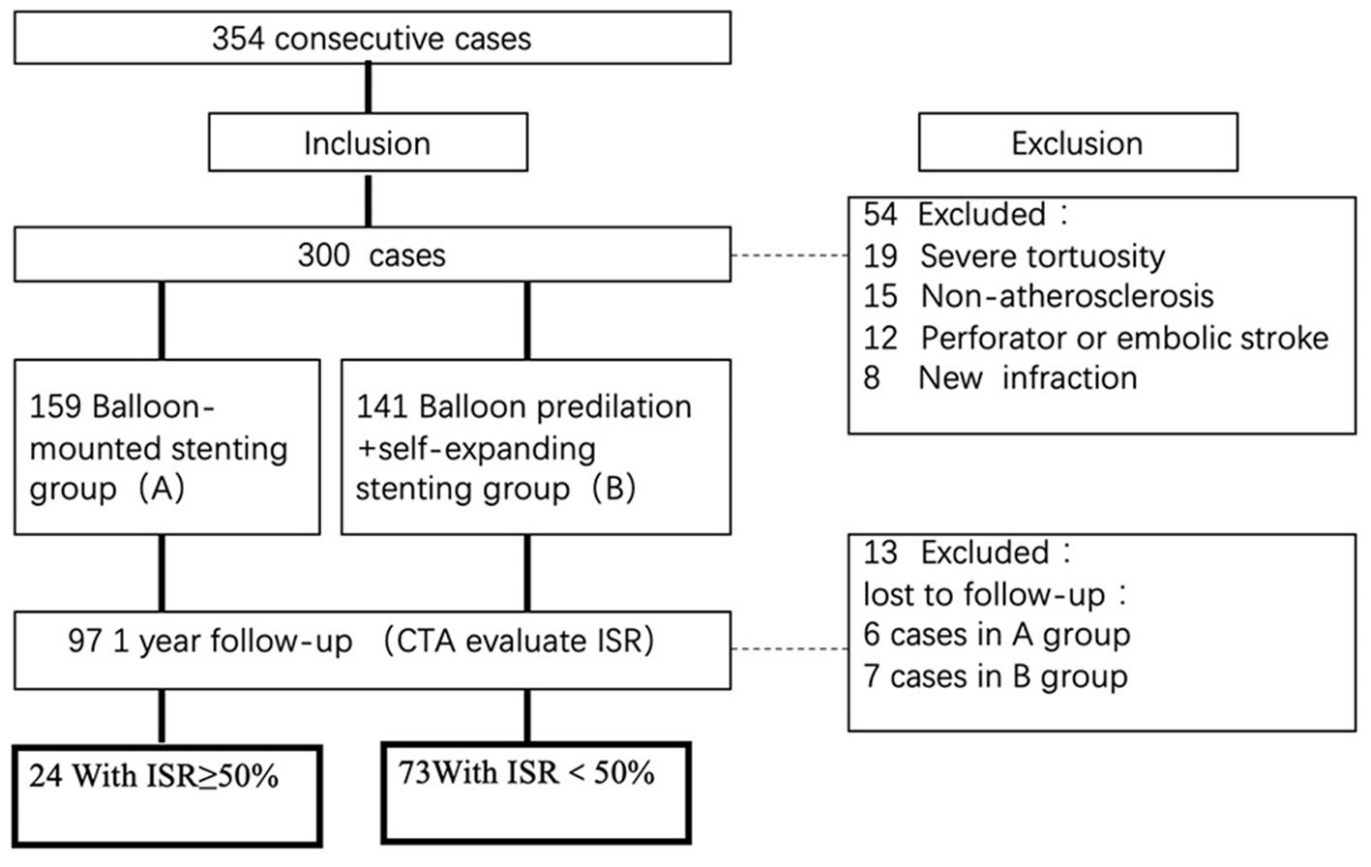
Flow chart of the study. Flow chart illustrating the numbers (n) of cases included and excluded in this analysis. ISR, in-stent restenosis; CTA, computed tomography angiography.

Patients were excluded if they had an acute ischemic stroke within 3 weeks, severe arterial tortuosity precluding the deployment of endovascular devices, non-atherosclerotic on an MRI, embolic or perforator stroke on MRI or CT, or a baseline modified Rankin Scale Score >3. Only patients without risk factors for intracranial atherosclerosis or patients with lesions suspected of being non-atherosclerotic by regular CT, MRI, or DSA had high-resolution MRI. All images were centrally reviewed by at least two radiologists who would also adjudicate any disagreement. All data were reviewed centrally by the executive committee to determine the patient's eligibility for enrollment. A flow chart illustrating the number (n) of cases included and excluded can be viewed in Figure 1.

### Endovascular Device and Procedure

Operators were instructed to choose the devices based on the following guidelines, also taking into consideration their experience and preference to ultimately select what they thought were best suited for the patients. For patients with smooth arterial access and Mori A lesions (13), the Apollo balloon-mounted stent (MicroPort Neuro Tech, Shanghai, China) was selected. For patients with tortuous arterial access, Mori C lesion, or a lesion with a significant mismatch in the diameter between the proximal and distal segments, balloon predilation plus self-expanding stent (Gateway balloon plus Wingspan stent system) (Stryker Neurovascular, Fremont, California, USA) was preferred.

The procedures were performed by experienced neurointerventionists at each participating site. Either general anesthesia or local anesthesia was chosen depending on the operators' experience and preference. Intravenous heparin was administered after the placement of vascular access using a bolus of 75 U/kg followed by half the dose 1 h later. The guiding catheter was advanced into the cervical vertebral or internal carotid artery as high as the vessel tortuosity allowed. Perioperative systolic blood pressure was kept between 100- and 120-mm Hg. Non-contrast head CT was obtained to exclude potential hemorrhage after the procedure. All patients were given a weight-based dose of 0.4–0.6-mL Fraxiparine (Sanofi Winthrop Industry) every 12 h subcutaneously for 3 days and monitored closely until discharge.

### Perioperative Management

All patients received aspirin (100 mg/d) and clopidogrel (75 mg/d) for >5 days before the operation or a loading dose of 300 mg clopidogrel if the procedure was considered urgent. All of the cases tested the ADP-induced platelet inhibition rate (ADP%). Based on the result of ADP%, we switched to P2Y12-receptor inhibitors. They were maintained on aspirin (100 mg/d) plus clopidogrel (75 mg/d) for 90 days after stenting. Aggressive medical therapy was implemented to achieve the following long-term goals: systolic blood pressure of <140 mm Hg (or <130 mm Hg in patients with diabetes mellitus), low-density lipoprotein of <70 mg/dL (1.81 mmol/L) or a decrease by 50%, diabetes control, smoking cessation, and lifestyle modification for obesity and sedentary state.

### Follow-Up

Follow-up information on clinical outcomes was collected and reviewed by trained personnel who were blinded to treatment assignment at study entry, the day of discharge, 30-days follow-up, and a face-to-face interview every 3 months. All follow-up visits were in person unless the patient could not return for the visit, in which case a telephone follow-up was completed. DSA or CT angiography (CTA) was obtained at the 12-months follow-up after the procedure. ISR was defined as a lesion demonstrating stenosis of >50% adjacent to the stent (i.e., within or immediately adjacent 5 mm) on follow-up imaging (12, 14).

### Statistical Analysis

Continuous variables, such as demographic, clinical, and characteristics of stenosis lesions and imaging findings, were described as the mean ± standard deviation (*SD*), and categorical variables were expressed as the frequency and percentage of the group. The median and inter-quartile range (IQR) were used to describe the univariable distribution, including admission NIHSS and mRS. Comparative analyses between the ISR ≥ 50% and ISR < 50% groups were analyzed using *T*-tests for continuous variables, Mann-Whitney *U* non-parametric tests for ordinal variables (NIHSS, mRS, hs-CRP, length of the stent), and chi-square tests for categorical variables. Binary logistic regression analyses were conducted to test the relationship between ISR and the variables including length of the stent, stent type, anesthesia mode, and Mori type. All statistical analyses, including crude and adjusted odds ratios and 95% confidence intervals (OR, 95% CI), were performed using SPSS software (IBM, SPSS Statistics 25.0, Armonk, New York, USA), and *P* < 0.05 were considered statistically significant.

## Result

### Baseline Characteristics

Of 354 consecutive cases who underwent endovascular treatment in the designated period time, 300 patients were recruited, including 159 patients treated with a balloon-mounted stent and 141 patients with balloon predilation plus self-expanding stent. Ninety-seven patients finally fulfilled the inclusion criteria and performed CTA images in a median of 12.7 months (IQR 11.0–15.1 months). Twenty-four cases were identified with ISR ≥ 50%, while 73 cases had ISR < 50%.

All patients were confirmed by DSA before the endovascular procedure, while CTA was confirmed in a 12-months follow-up in 97 patients. These patients had an average age of 57.12 years (SD 10.53), and male sex accounted for 77.32%. There was no significant difference between the two groups concerning the risk factors, admission features, and blood tests, except for hs-CRP levels. Lesions in 45 cases were located at the anterior circulation, including intracranial internal carotid artery (12 cases) and middle cerebral artery (33 cases), while the other 52 cases were located at the posterior circulation, including intracranial vertebral artery (23 cases) and basilar artery (29 cases). The mean length of the lesion and stenosis degree in both groups had no statistical significance. At the 12-months follow-up, TIA and cerebral infarction in ISR ≥ 50% and ISR < 50% were 8/72 (11.1%) and 4/24 (16.7%), respectively (*p* = 0.704). Baseline demographic and clinical characteristics are displayed in Table 1.

**Table 1:** Baseline demographic and clinical characteristics.

**Variables**	**All (*n* = 97)**	**Patients with ISR ≥ 50% (*n* = 24)**	**Patients with ISR < 50% (*n* = 73)**	***P*-value**
**Demographics**				
Age, years, mean (*SD*)	57.12 (10.53)	59.21 (9.57)	56.44 (10.80)	0.266
Male sex	75 (77.32%)	18 (75.00%)	57(78.08%)	0.754
**Risk factors**				
Hypertension	27 (27.8%)	17 (70.8%)	53 (72.6%)	0.867
Diabetes mellitus	31 (32.0%)	9 (37.5%)	22 (30.1%)	0.502
Hypercholesterolemia	37 (38.1%)	10 (41.7%)	27 (37.0%)	0.682
Current smoking	28 (28.9%)	6 (25.0%)	22 (30.1%)	0.153
**Features on admission**				
BMI, mean (*SD*)	25.6 (2.9)	25.9 (2.6)	25.5 (3.0)	0.601
Systolic BP, mmHg (*SD*)	137 (18)	139 (18)	137 (18)	0.670
Diastolic BP, mmHg (*SD*)	81 (11)	81 (11)	81 (11)	0.995
Glucose, mmol/L (*SD*)	6.4 (3.5)	6.8 (2.7)	6.3 (3.8)	0.559
LDL-C, mmol/L (*SD*)	2.3 (0.9)	2.5 (1.0)	2.3 (0.9)	0.231
hs-CRP (mg/L)^a^	4.0 (6.8)	8.2 (11.4)	2.8 (4.1)	0.032
NIHSS score	0.0 (0–1.5)	0.0 (0.0–1.8)	0.0 (0.0–1.5)	0.403
mRS score	1.0 (0.0–1.0)	1.0 (0.0–1.0)	1.0 (0.0–1)	0.538
**Lesion**				
Anterior circulation	45 (46.4%)	11 (45.8%)	34 (46.6%)	0.950
Posterior circulation	52 (53.6%)	13 (54.2%)	39 (53.4%)	
Length of lesion (mm)	7.8 (3.0)	8.1 (2.9)	7.8 (3.0)	0.675
Degree of stenosis (%)	85.2 (6.6)	84.9 (7.1)	85.3 (6.4)	0.791

*ISR, in-stenting restenosis; BMI, body mass index; LDL-C, low density-lipoprotein cholesterol; hs-CRP, high-sensitivity C-reactive protein; NIHSS, National Institutes of Health Stroke Scale; mRS, modified Rankin Scale*.

a *31 missing values*.

### Procedural Features and Treatment

Among these patients, the incidence of ISR ≥ 50% was higher in patients who underwent local anesthesia than in those who underwent general anesthesia (11/23 vs. 12/74, *P* = 0.003). The rate of ISR ≥ 50% was significantly higher in patients who underwent balloon-mounted stents than in those who underwent balloon-predilation plus self-expanding stents (19/57 vs. 5/40, *p* = 0.019). The mean stent lengths were longer in the ISR ≥ 50% group than in the ISR < 50% group (11.0 ± 3.9 vs. 12.8 ± 3.4, *p* = 0.020). Mori type, residual stenosis rate, or primary outcome at the 12-months follow-up did not have a significant intergroup difference. Details of the interventional procedure results are displayed in Table 2.

**Table 2:** Comparison of interventional procedural results stratified by ISR.

**Variables**	**All (*n* = 97)**	**Patients with ISR ≥ 50% (*n* = 24)**	**Patients with ISR < 50% (*n* = 73)**	***P*-value**
**Mori type**				0.229
A	24 (24.7%)	3 (12.5%)	21 (28.8%)	0.109
B	54 (55.7%)	15 (62.5%)	39 (53.4%)	0.438
C	19 (19.6%)	6 (25.0%)	13 (17.8%)	0.636
**Mode of anesthesia**				0.003
General anesthesia	74 (76.3%)	13 (54.2%)	61 (83.6%)	
Local anesthesia	23 (23.7%)	11 (45.8%)	12 (16.4%)	
**Stent type**				0.019
Wingspan	40 (41.2%)	5 (20.8%)	35 (47.9%)	
Apollo	57 (60.6%)	19 (79.2%)	38 (52.1%)	
Length of stent, *SD* (mm)	12.3 (3.6)	12.8 (3.4)	11.0 (3.9)	0.020
Residual stenosis, *SD* (%)	8.6 (8.1)	7.7 (10.2)	8.9 (7.3)	0.519
**Events**				
Composite endpoint^a^	8 (8.2%)	2 (8.3%)	6 (8.2%)	1.000
Any stroke	3 (3.1%)	0 (0.0)	3 (2.3%)	0.572
Ischemic stroke	3 (10.2)	0 (0.0)	3 (4.1%)	0.188

*ISR, in-stenting restenosis*.

a *The composite endpoint includes ischemic stroke within the territory of the target vessel, hemorrhagic stroke and vascular death*.

### Risk Factors Associated With ISR

Binary logistic regression analysis of predictors for the longer stent (adjusted OR 0.816, 95% CI 0.699–0.953; *p* = 0.010), balloon-mounted stent (adjusted OR 5.748, 95% CI 1.533–21.546; *p* = 0.009), or local anesthesia (adjusted OR 6.000, 95% CI 1.693–21.262; *p* = 0.006) were significantly associated with in-stent restenosis. The results of the binary logistic regression analysis are shown in Table 3.

**Table 3:** Binary logistic regression analysis of the association between risk factors and ISR.

**Variables**	**cOR**	**95% CI**	***P*-value**	**aOR**	**95% CI**	***P*-value**
Length of stenosis	1.034	0.887–1.204	0.671	1.018	0.849–1.220	0.847
Length of stent	0.872	0.761–0.998	0.046	0.816	0.699–0.953	0.010
Stent type	3.500	1.180–10.378	0.024	5.748	1.533–21.546	0.009
Anesthesia mode	4.301	1.561–11.885	0.005	6.000	1.693–21.262	0.006

*ISR, in-stenting restenosis; cOR, crude odds ratio; aOR, adjust odds ratio*.

## Discussion

In recent evidence, the WEAVE study demonstrated that the incidence of the peri-procedural event was only 2.6% because of on-label stent use for ICAS patients (5). In this year, the Wingspan One-Year Vascular Events and Neurologic Outcomes (WOVEN) trial that consequently studied the WEAVE trial presented in 2020 ISC. A total of 107 of 129 imaging follow-up results showed that 16.8% of patients had restenosis of 70% or greater in the mean time of 5 months (range 1–11 months) (15). In our study, the rate of in-stent restenosis is higher than in the WOVEN trial. This could be due to several reasons: (1) There were different ISR definitions (≥70% in the WOVEN trial vs. > 50% in our study); (2) The different patterns of images in the WOVEN trial included TCD, CTA, MRA, and DSA, while there was uniform CTA follow-up in our study; (3) The mean time of imaging follow-up in WOVEN was shorter (5 vs. 12.7 months).

Generally, the conventional predictors of in-stent restenosis of ICAS included risk factors of stroke and endovascular procedure related factors. To our knowledge, our study includes the largest sample to analyze endovascular procedure risk factors for in-stent stenosis for ICAS with uniform follow-up images in prospective controlled trials, although patients were retrospectively analyzed from a prospectively collected database in multiple centers. According to the analysis of the level of hs-CRP, characteristics of the intracranial stenosis lesion, mode of anesthesia, and features of the stent in the endovascular procedure, we tried to identify the risk factors for ISR of endovascular treatment in intracranial atherosclerotic stenosis.

### The Type and Length of the Stent

In our controlled study, the rate of ISR ≥ 50% was significantly higher in the balloon-mounted stent group than in the self-expandable stent group (33.3 vs. 12.5%). The rate of restenosis was slightly higher than that in our earlier study (20.3%) with coronary artery stents and the VISSIT study (26.5%) (4, 16), but was similar to Jin's study, which showed that the restenosis rate with the Apollo stent was 27.5% (24/87) vs. the Wingspan, which was 24.6% (17/69). In a recent study, Baik et al. reported insertion of a balloon-expandable stent (BES) with symptomatic middle cerebral artery stenosis, and the overall incidence of restenosis or reocclusion was 14.7% (5/34) with long-term follow-up (17). We concluded that 19 patients presented restenosis by performing balloon-mounted stents, including seven cases with basilar artery stenosis and four cases with intracranial vertebral artery stenosis. Therefore, in-stent restenosis of endovascular treatment for stenosis with this type of stent could be associated with the location of lesions, particularly in the posterior circulation.

In our study, the length of the stent, but not lesion length or residual stenosis, was a predictor of in-stent restenosis. Albuquerque reported that Wingspan-related ISR typically occurs as a focal shorter lesion, but more than a quarter of cases (26.8%) developed 50% of the length of the stented segment restenosis. The longer stent implantation, the more likely the ISR is to occur (18). We hypothesized that longer stents are associated with endothelial dysplasia, inflammatory responses, and the formation of new plaques, thus accelerating in-stent restenosis. In our further analysis, the difference in length between the self-expanding stent group and the balloon-expandable stent group was not significantly different (12.00 ± 3.62 mm vs. 8.40 ± 0.55 mm, *P* = 0.088).

With the development of new neurointerventional devices in recent years, stents delivered *via* microcatheters and assisted aneurysm embolization have increasingly been performed in ICAS. The report from Feng et al. supported the use of the more flexible Enterprise stent (Codman & Shurtleff, Raynham, Massachusetts, USA) for complex symptomatic ICAS, i.e., tortuous vessel pathways, longer than 15 mm stenosis lesions, or arterial bifurcation. Except for the feasibility and safety of this stent used for ICAS in the small sample observed study, only three cases (6.81%) presented >50% ISR compared to 86.4% (38/42) of patients who underwent a mean 22-months period of image follow-up (19). The drug-eluting balloon or stent has been used for stenosis of the coronary artery, and several observational studies have focused on the effectiveness and safety of endovascular treatment for ICAS. According to a systematic review and meta-analysis of the drug-eluting stent or drug-eluting balloon predilation with wingspan stent for ICAS studies, the ISR rate is 4.1 and 13%, respectively, which is lower than our study (20, 21). These studies are not high-level recommendations, but may be used to advance endovascular therapy and reduce the ISR rate in long-term image follow-up.

There is a paradox in our result that the complication rate of stroke in patients with ISR ≥ 50% is lower than that in patients with ISR < 50%. There was no statistical significance in the incidence of complications of stroke or ischemic stroke in both groups. We identified a downstream artery embolism due to in-stent intraplaque hemorrhage in a patient with ISR < 50% by HR-MRI. However, other reasons for symptomatic ISR in patients with ICAS need to be further investigated. Additional clinical and radiographic long-term follow-up cases could clarify the association between ISR and ischemic stroke.

### Modality of Anesthesia

Management of anesthesia in the endovascular procedure for non-acute stroke patients with ICAS was not discussed in recent literature. Although more RCTs or other trials have been published since 2015 on mechanical thrombectomy for acute stroke with large vessel occlusion, the feasibility and safety of any type of anesthesia, including general anesthesia, conscious sedation, or local anesthesia, are currently in doubt (22, 23). According to this study, ISR in groups between patients who underwent intubation and local anesthesia was 17.6%, 13/74, vs. 33.3%, 11/33, *P* = 0.003, respectively. Local anesthesia is easy to perform during the operation procedure, offering the advantages of being low in cost, being less time consuming, and allowing for earlier detection of patient detoriation (24). However, general anesthesia could minimize patient movement during the procedure, perform plentiful submaximal inflation, and reduce complications of technical operations, such as iatrogenic perforation or dissection.

### hs-CRP

Hs-CRP, as an inflammatory biomarker, has been proven to be associated with ISR in patients implanted with coronary or carotid artery stents (25, 26). Moreover, high CRP levels are a predictor of the asymmetric growth of restenotic tissue because of the differential distribution of shear stress and its effect on neointimal tissue shape mediated by the inflammatory process (27). Our analysis suggested a significant association between elevated serum hs-CRP and ISR. However, limited samples need to be expanded in further studies.

### Limitation

Finally, our study has some limitations. We retrospectively reviewed the large multicenter, prospective, observative database which only allows us to suggest association rather than causation. Based on the characteristics of ICAS lesions and operators' experience or preference, interventionists individually selected a balloon-mounted stent or balloon plus a self-expanding stent. It is difficult to eliminate selection bias in the allocation of the two groups accurately. Second, despite analysis as a predictor of different locations in anterior or posterior circulation of ISR, details of different segments of the intracranial artery that underwent endovascular treatment may be a predictor of ISR. Third, 95% CI for balloon-mounted stents and local anesthesia is very large because of our retrospective analysis of available information, and this may be subject to selective bias. The analysis result needs to be interpreted cautiously. Furthermore, based on our data, 13 patients performed both follow-up CTA and DSA examinations (the consistent rate of CTA and DSA results was statistically tested to be 84.6%). However, CTA was used for the evaluation of ISR as a uniform modality of follow-up image in our study, but DSA was not, because generally patients were reluctant to accept more convenient and less invasive imaging procedures.

## Conclusion

Our study suggested that a higher hs-CRP level, longer stents, balloon-mounted stents implanted in the intracranial vertebral or basilar artery, and local anesthesia were significantly associated with in-stent restenosis. Owing to advances in this new era of neuro-endovascular devices, technology, and neuroradiology, further studies are required to identify novel and accurate biomarkers or image markers associated with ISR in ICAS patients.

## Data Availability Statement

The original contributions presented in the study are included in the article/supplementary materials, further inquiries can be directed to the corresponding author/s.

## Ethics Statement

The studies involving human participants were reviewed and approved by ethics committee of Beijing Tiantan Hospital. The patients/participants provided their written informed consent to participate in this study.

## Author Contributions

XG: study design, literature search, data collection, database establishment, and chief writer of this manuscript. NM, FG, and D-PM: data analysis. GL: manuscript reviewing, modification, and data analysis. Z-RM: study design, chief writer of this manuscript, and guarantor. All authors contributed to the article and approved the submitted version.

## Conflict of Interest

The authors declare that the research was conducted in the absence of any commercial or financial relationships that could be construed asa potential conflict of interest.
