# Corrigendum: Long-Term Risk Factors for Intracranial In-Stent Restenosis From a Multicenter Trial of Stenting for Symptomatic Intracranial Artery Stenosis Registry in China

**DOI:** 10.3389/fneur.2021.673264

**Published:** 2021-03-30

**Authors:** Xu Guo, Ning Ma, Feng Gao, Da-Peng Mo, Gang Luo, Zhong-Rong Miao

**Affiliations:** Department of Interventional Neuroradiology, Beijing Tiantan Hospital, Capital Medical University, Beijing, China

**Keywords:** cerebrovascular disease, stroke, endovascular treatment, interventional neurology, intracranial in-stent restenosis

In the original article, there were errors. Three numbers were incorrectly added due to mistaken data import from SPSS into the submitted manuscript. A correction has been made to *Discussion, The Type and Length of the Stent, Paragraph 1*. The corrected paragraph is shown below.

In our controlled study, the rate of ISR ≥ 50% was significantly higher in the balloon-mounted stent group than in the self-expandable stent group (33.3 vs. 12.5%). The rate of restenosis was slightly higher than that in our earlier study (20.3%) with coronary artery stents and the VISSIT study (26.5%) (4, 16), but was similar to Jin's study, which showed that the restenosis rate with the Apollo stent was 27.5% (24/87) vs. the Wingspan, which was 24.6% (17/69). In a recent study, Baik et al. reported insertion of a balloon-expandable stent (BES) with symptomatic middle cerebral artery stenosis, and the overall incidence of restenosis or reocclusion was 14.7% (5/34) with long-term follow-up (17). We concluded that 19 patients presented restenosis by performing balloon-mounted stents, including seven cases with basilar artery stenosis and four cases with intracranial vertebral artery stenosis. Therefore, in-stent restenosis of endovascular treatment for stenosis with this type of stent could be associated with the location of lesions, particularly in the posterior circulation.

The authors apologize for these errors and state that they do not change the scientific conclusions of the article in any way. The original article has been updated.

